# Association of Infectious Disease Consultation With Clinical Outcomes in Patients With *Staphylococcus aureus* Bacteremia at Low Risk for Endocarditis

**DOI:** 10.1093/ofid/ofy142

**Published:** 2018-06-14

**Authors:** Anna Yousaf, Grayson L Baird, Leonard Mermel

**Affiliations:** 1 Warren Alpert Medical School of Brown University, Providence, Rhode Island; 2 Division of Infectious Diseases, Rhode Island Hospital, Providence, Rhose Island; 3 Lifespan Biostatistics Core, Rhode Island Hospital, Providence, Rhose Island; 4 Department of Epidemiology and Infection Control, Rhode Island Hospital, Providence, Rhose Island

**Keywords:** infectious disease consult, *Staphylococcus aureus*

## Abstract

Infectious disease (ID) consultation in patients with *Staphylococcus aureus* bacteremia who were at low risk for endocarditis and who had no secondary site of infection was associated with a longer course of antibiotics (median duration of intravenous antimicrobial therapy of 31 days and 15 days in those with and without ID consultation, respectively; *P* ≤ .01), and based on Kaplan-Meier survival analysis, reduced in-hospital mortality (*P* = .2), and reduced 30-day mortality after discharge (*P* = .4). ID consultation was also associated with a higher readmission rate within 90 days of discharge: 46% and 34% with and without ID consultation, respectively (*P* = .2).

Infectious diseases (ID) consultation in patients with *Staphylococcus aureus* bacteremia (SAB) is associated with a decrease in 30-day mortality, 90-day mortality, length of stay, SAB relapse rates, and more frequent adherence to standards of care (antibiotic choice, antibiotic duration, and follow-up blood cultures) [1, 2]. Multiple guidelines have been published on the management of *S. aureus* infections [3, 4] and adherence to these guidelines decreases 30-day mortality [5, 6]. Despite these guidelines and their associated benefits, management of *S. aureus* bacteremia remains variable [7]; although some institutions mandate ID consultation for all patients with SAB, others do not. This raises the questions of whether there is a subpopulation of patients with SAB who do not require ID consultation for better outcomes and whether clinical criteria used to identify this patient group could be used to triage patients with SAB. The aim of this study is to examine the relationship between ID consultation and outcomes in patients with *S. aureus* bacteremia at low risk for endocarditis and who have no apparent secondary sites of infection [8–10]. It is hypothesized that those who received ID consultation would have better outcomes than those patients who did not.

## METHODS

### Study Setting and Sample

This was a retrospective cohort study including patients admitted to any of the 3 hospitals in our health care system: Rhode Island Hospital, Newport Hospital, and The Miriam Hospital, licensed for 719, 129, and 247 beds, respectively, and who had positive blood cultures for *S. aureus* from January 1, 2012, to December 31, 2016. TheraDoc software (Salt Lake City, UT) was used to identify patients. Approval was obtained from the Lifespan Institutional Review Board before retrospective chart review.

### Definitions

Adult patients were excluded if they had any of 6 risk factors for endocarditis: community-acquired bacteremia (ie, first blood culture growing *S. aureus* drawn within 48 hours of presentation to the emergency department), bacteremia documented 96 or more hours after the first positive blood culture was drawn, presence of a permanent intracardiac device other than a coronary stent, hemodialysis dependence (ie, dialysis through an arteriovenous fistula or graft, or long-term tunneled dialysis catheter), secondary foci of infection (ie, any infection presumed to be due to hematogenous spread), or stigmata of endocarditis (ie, a new murmur, new petechia, or nodules on the extremities) [8–10]. Patients were also excluded if duration of bacteremia was unknown (ie, the patient did not have a negative blood culture drawn during the 96 hours after the first positive blood culture was drawn, or they did not have any repeat blood cultures after the first positive blood culture was drawn). Patients were excluded if their only positive blood culture was drawn from a central venous catheter and percutaneously drawn blood cultures were negative. Patients were counted only once. Patients who were hemodialyzed using a temporary, nontunneled dialysis catheter were included (ie, intensive care unit [ICU] patients briefly hemodialyzed and who had their dialysis catheter removed before hospital discharge).

Primary outcomes were in-hospital mortality, 30-day mortality starting after hospital discharge, length of stay starting from the day the first positive blood culture was drawn, ICU length of stay starting from the day the first positive blood culture was drawn, and readmission within 90 days of the discharge date. Secondary outcomes were duration of intravenous antimicrobial therapy, inappropriate therapy, and removal of a central venous catheter when central venous catheter–related bloodstream infection (CVCRBSI) was suspected. Appropriate therapy for methicillin-susceptible *S. aureus* (MSSA) was defined as intravenous beta-lactam therapy, and for methicillin-resistant *S. aureus* (MRSA), it was defined as intravenous daptomycin or vancomycin and oral or intravenous linezolid. Inappropriate therapy was defined as non-beta-lactam therapy for MSSA or no antibiotic therapy for MRSA or MSSA. CVCRBSI was defined as a positive blood culture drawn from the central venous catheter with a positive percutaneously drawn blood culture and no other identified source of bacteremia. Short-term peripheral venous catheter–related bloodstream infection was defined as phlebitis or purulent discharge at a peripheral venous catheter insertion site and growth of *S. aureus* in peripheral blood cultures.

### Data Analysis

Group comparisons were made using a 1-tailed *t* test and Fisher exact test. Time-to-event analyses were performed using Kaplan-Meier estimation with SAS Software (SAS Inc., Cary, NC) using the LIFETEST procedure, where last known follow-up was used for censoring.

## RESULTS

**Figure 1. Flow diagram illustrating the selection of study cohort.**
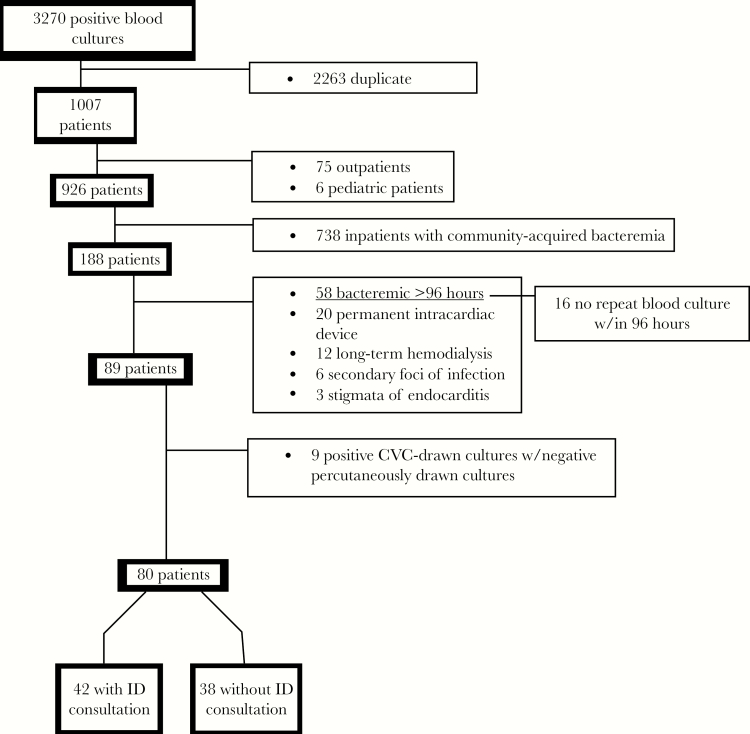


Eighty patients were included in the study (Figure 1). The majority of this “low-risk” group was comprised of patients with transient *S. aureus* bacteremia due to peripheral or central venous catheter infection. Patients receiving an ID consult were older (median age, 67 vs 57 years; *P* = .04), had more coronary artery disease (19% [8/42] vs 11% [4/38], *P* = .3), and more had chronic kidney disease stages 3–5 (13% [5/42] vs 5% [2/38], *P* = .3) (Table 1). The most commonly identified sources of bacteremia were central venous catheter infections and peripheral venous catheter infections, each found in 26% (21/80) of patients. Three percent (2/80) of patients had superficial surgical site infections which were surgically debrided during the index admission, another 3% had abscesses which were drained during the index admission, and 1% (1/80) of patients had a colovesicular fistula which was not surgically repaired (Table 2).

**Table 1.** Patient Characteristics

	With ID Consult (n = 42)	Without ID Consult (n = 38)	*P* Value
Age, median (min–max), y	67 (18–99)	57 (20–91)	.04
Female	48% (20/42)	39% (15/38)	.3
Comorbidities			
Active malignancy	10% (4/42)	18% (7/38)	.2
Chronic kidney disease stage 1–2	2% (1/42)	3% (1/38)	.7
Chronic kidney disease stage 3–5	13% (5/42)	5% (2/38)	.3
Cirrhosis	5% (2/42)	5% (2/38)	.7
Coronary arterial disease	19% (8/42)	11% (4/38)	.3
Corticosteroid use >14 d	2% (1/42)	0% (0/38)	
Diabetes mellitus	21% (9/42)	26% (10/38)	.4
Heart failure with reduced ejection fraction	7% (3/42)	5% (2/38)	.5
HIV infection	2% (1/42)	0% (0/38)	
Intravenous drug abuse	2% (1/42)	0% (0/38)	

Abbreviation: ID, infectious disease.

**Table 2.** Type and Source of Staphylococcus aureus Bacteremia

	With ID Consult (n = 42)	Without ID Consult (n = 38)	*P* Value
Methicillin-susceptible *S. aureus* bacteremia	81% (34/42)	66% (25/38)	.1
Methicillin-resistant *S. aureus* bacteremia	19% (8/42)	34% (13/38)	.1
Source of bacteremia			
CVCRBSI^a^	26% (11/42)	26% (10/38)	.6
PVCRBSI^b^	38% (16/42)	13% (5/38)	.01
Pneumonia	0% (0/42)	16% (6/38)	
Unknown	31% (13/42)	37% (14/38)	.4
Other	5% (2/42) 1 = colovesicular fistula/urine 1 = surgical site infection	8% (3/38) 1 = surgical site infection 1 = intra-abdominal abscess 1 = chest wall abscess	.4

Abbreviations: CVCRBSI, central venous catheter–related bloodstream infection; ID, infectious disease; PVCRBSI, peripheral venous catheter–related bloodstream infection.

^a^Central venous catheter–related bloodstream infection.

^b^Peripheral venous catheter–related bloodstream infection.

**Figure 2.**
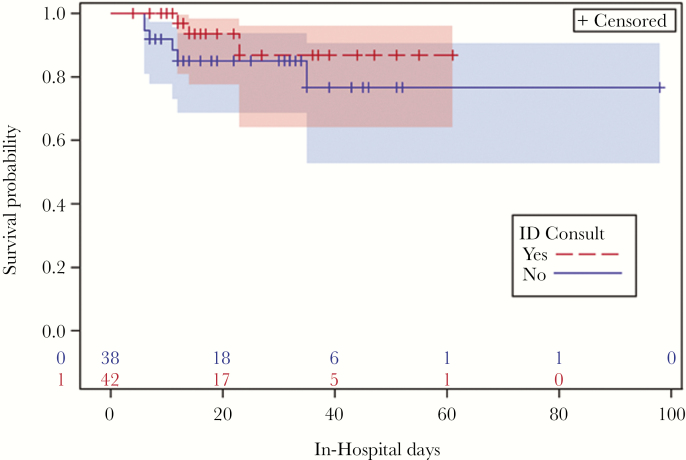


Survival analysis revealed in-hospital mortality to be 14.9% at 12 days after admission for those without ID consultation and 3.1% at 12 days after admission for those with ID consultation (*P* = .2) (Table 3). In addition, mortality 33 days after discharge was 10.3% for those without ID consultation, and mortality 75 days after discharge was 5.6% for those with ID consultation (Figure 2). The median time to these events could not be calculated because 50% of patients did not die in the hospital or after discharge. Three patients in each group were lost to follow-up after the index hospitalization (ie, the last patient contact was at hospital discharge). The cause of death was directly related to *S. aureus* infection in 60% (3/5) of patients who had ID consultation and 67% (6/9) of patients who did not have ID consultation (Tables A1 and A2). Readmission within 90 days of discharge was 46% (18/39) for those patients with ID consultation and 34% (11/32) for patients without ID consultation, (*P* = .2). The reasons for readmission in the patients with ID consultation were fever, peripherally inserted central catheter (PICC) complications, and drug toxicity in 28% (5/18), 0%, and 11% (2/18) of cases, respectively, and 27% (3/11), 9% (1/11), and 0% of cases, respectively, in the patients without ID consultation. Of the patients with ID consultation who were readmitted, 67% (12/18) had repeat blood cultures that were negative; 33% (6/18) did not have repeat blood cultures. Of the patients without ID consultation who were readmitted, 64% (7/11) had repeat blood cultures that were negative; 36% (4/11) did not have repeat blood cultures. All patients with ID consultation readmitted for fever had negative repeat blood cultures, 66% (2/3) of patients without ID consultation readmitted for fever had negative repeat blood cultures, and 1 patient did not have repeat blood cultures.

The median duration of intravenous antimicrobial therapy in patients who had an ID consult was 31 days, compared with 15 days in those without an ID consult (*P* ≤ .01) (Table 3). In the patients with ID consultation, the most common intravenous antibiotics at the time the first positive blood culture was obtained were vancomycin (38% [16/42]) or a beta-lactam antibiotic (38% [16/42]) (Table 4). In the patients without ID consultation, beta-lactam antibiotics were the most common antibiotics being administered at the time the first positive blood culture was obtained (34% [13/38]). Patients with ID consultation were more likely to be on IV vancomycin at the time the first positive blood culture was obtained compared with patients without ID consultation (38% [16/42] vs 24% [9/38], *P* = .1). For patients with ID consultation, the antibiotic was changed 62% of the time. This primarily involved discontinuing vancomycin and recommending nafcillin or cefazolin in cases of methicillin-susceptible *S. aureus* bacteremia.

**Table 3.** Clinical Outcomes

	With ID Consult (n = 42)	Without ID Consult (n = 38)	*P* Value
Length of stay from 1st positive blood culture, median (IQR), d	11 (8–19)	11 (5–23)	.4
Length of stay in ICU from 1st positive blood culture, median (IQR), d	13 (5–13)	10 (6–20)	.4
Duration of IV antimicrobial therapy, median (IQR), d	31 (17–32)	15 (8–29)	<.01
In-hospital mortality at 12 d	3.1%	14.9%	.2
Mortality at 75 and 33 d after discharge, respectively^a^	5.6%	10.3%	.4
Cause of death related to SAB^b^	40% (2/5)	67% (6/9)	.3
Readmission within 90 d of discharge	46% (18/39)	34% (11/32)	.2
Reason for readmission			
Fever	28% (5/18)	27% (3/11)	.6
PICC complication	0% (0/18)	9% (1/11)	
Drug toxicity	11% (2/18)	0% (0/11)	

Abbreviations: ICU, intensive care unit; ID, infectious disease; IQR, interquartile range; IV, intravenous; PICC, peripherally inserted central catheter; SAB, *Staphylococcus aureus* bacteremia.

^a^Three patients from each group were lost to follow-up after discharge.

^b^ *Staphylococcus aureus* bacteremia (see the Appendix for cause of death).

**Table 4.** SAB Management

	With ID Consult (n = 42)	Without ID Consult (n = 38)	*P* Value
Antimicrobial choice (at time 1st positive blood culture obtained)			
Beta-lactam	38% (16/42)	34% (13/38)	.4
Vancomycin	38% (16/42)	24% (9/38)	.1
Beta-lactam + vancomycin	19% (8/42)	26% (10/38)	.3
Linezolid	0% (0/42)	3% (1/38)	
Daptomycin	0% (0/42)	0% (0/38)	
No treatment	0% (0/42)	3% (1/38)	
Other	5% (2/42) vancomycin + ciprofloxacin (1/42) vancomycin + meropenem (1/42)	11% (4/38) ciprofloxacin (2/38) vancomycin + meropenem (1/38) vancomycin + aztreonam (1/38)	.3
Central venous catheter removed for suspected CVCRBSI	100% (11/11)	100% (10/10)	.99
TTE^a^ obtained	57% (24/42)	47% (18/38)	.3
TEE^b^ obtained	2% (1/42)	3% (1/38)	.7
TTE and TEE obtained	38% (16/42)	5% (2/38)	<.01
Echocardiogram showing endocarditis	0% (0/41)	0% (0/21)	

Abbreviations: CVCRBSI, central venous catheter–related bloodstream infection; ICU, intensive care unit; ID, infectious disease; TEE, transesophageal echocardiogram; TTE, transthoracic echocardiogram.

^a^Transthoracic echocardiogram.

^b^Transesophageal echocardiogram.

^c^All antimicrobial agents administered intravenously except for ciprofloxacin and linezolid.

In the patients without ID consult, 11% (4/38) received inappropriate therapy: ciprofloxacin for MSSA bacteremia in 2 patients, meropenem for MSSA bacteremia in 1 patient, and no antibiotic treatment in 1 bacteremic patient. For the 2 patients who received ciprofloxacin, 1 was alive 90 days after discharge, and the other was readmitted with recurrent intra-abdominal abscess. The patient who did not receive any antibiotic treatment was considered by their care team to have contaminated percutaneously drawn blood cultures. This patient was discharged and lost to follow-up. In the patients with ID consult, 2% (1/42) received potentially inappropriate although adequate therapy: meropenem for MSSA bacteremia in a patient with postoperative MSSA ventriculitis and a penicillin allergy. Of note, central venous catheters were removed in all cases of CVCRBSI in both groups.

In patients with ID consultation, 98% (41/42) had an echocardiogram: 57% (24/42) received a transthoracic echocardiogram (TTE) alone, 2% (1/42) received a transesophageal echocardiogram (TEE) alone, and 38% (16/42) received a TTE followed by a TEE. In these patients, the echocardiogram was performed after it was recommended by the ID consultant in 69% of patients (29/42). In patients without ID consultation, 55% (21/38) had an echocardiogram: 47% (18/38) received a TTE alone, 3% (1/38) had a TEE alone, and 5% (2/38) received a TTE followed by a TEE. None of the TTE or TEE findings were consistent with a diagnosis of infective endocarditis (Table 4).

Our hospital system introduced an antibiotic stewardship program in October 2015. The program reviews management of patients with *S. aureus* identified in blood cultures by polymerase chain reaction. Eleven of the 80 patients in our study were hospitalized after this program was implemented (Table 5); none of these patients had a recommendation for ID consultation from the antibiotic stewardship program noted in the medical record; however, 9 of these patients already had ID consultation.

**Table 5.** Admission Service and Admission Year

	With ID Consult (n = 42)	Without ID Consult (n = 38)	*P* Value
Admission service			.9
Medical ICU	19% (8/42)	8% (3/38)	
Surgical ICU	0% (0/42)	5% (2/38)	
Neurosurgical ICU	10% (4/42)	16% (6/38)	
Trauma ICU	2% (1/42)	5% (2/38)	
Cardiac ICU	10% (4/42)	8% (3/38)	
General medicine/neurology	57% (24/42)	53% (20/38)	
Other	2% (1/42)^a^	5% (2/38)^b^	
Admission year			
2012	29% (12/42)	21% (8/38)	.3
2013	14% (6/42)	29% (11/38)	.1
2014	24% (10/42)	34% (13/38)	.6
2015	14% (6/42)	13% (5/38)	.6
2016	19% (8/42)	3% (1/38)	<.01

Abbreviations: ICU, intensive care unit; ID, infectious disease.

^a^Orthopedic surgery.

^b^General surgery.

## DISCUSSION

Prior studies have assessed the impact of ID consultation on outcomes in patients with *S. aureus* bacteremia [1, 2], but to the best of our knowledge, they did not specifically assess patients at low risk for endocarditis [8–10] with no known secondary sites of infection. We found few patients who met preestablished, low-risk criteria, such that 926 patients were assessed and only 80 were evaluable. The majority of evaluable patients had transient *S. aureus* bacteremia predominantly arising from a central venous or short-term peripheral venous catheter. Short-term peripheral venous catheters are known to make up a significant portion of nosocomial bloodstream infections [11], and in our study, we found that they comprised 26% of *S. aureus* bacteremias. For patients with *S. aureus* bacteremia and without stratification regarding risk of endocarditis, ID consultation has been associated with a 52% reduction in total 30-day mortality (ie, including in-hospital and postdischarge mortality within 30 days) [1]. Infectious disease consultation was also associated with reduced mortality in our cohort of patients at low risk of endocarditis. However, we had limited power to demonstrate a significant difference. One patient without ID consultation died 24 hours after *S. aureus* was identified in blood cultures. All other patients without ID consultation who died did so more than 24 hours after their blood culture results became available to the care teams, making survivorship bias in the ID consult group less likely.

 Readmission within 90 days of discharge was higher in patients who received ID consultation, although this difference was not significant. The majority of these readmissions were due to underlying medical problems, rather than drug toxicity or PICC complications.

We found that patients in our institution who received ID consultation were more likely to have an echocardiogram despite the fact that they had a low pretest probability for endocarditis. No echocardiograms had findings consistent with a diagnosis of infective endocarditis. Thus, echocardiogram orders may be an opportunity for diagnostic stewardship when ID consultation is performed in such patients.

An additional area of concern at our institution is the fact that 28 patients were excluded from the study because they had an unknown duration of bacteremia: 16 of these patients did not have repeat blood cultures drawn within 96 hours of the first positive blood culture; 12 of these patients did not have any repeat blood cultures. Thus, continued bacteremia or clearance was not documented, in contrast to published guidelines [3, 4]. It is likely that ID consultation would assist in this regard.

A major limitation of this study is the retrospective design. Specifically, any differences observed between those with and without ID consultation could be due to the benefits of ID consultation, or lack thereof, as well as selection bias leading to ID consultation. For example, those receiving an ID consult could have been selected for ID consultation due to more severe infection or because of their comorbidities. The significant differences observed between the two groups were longer duration of intravenous antimicrobial therapy (4 weeks vs 2 weeks) and greater number of echocardiograms obtained in patients receiving ID consultation. The majority of these echocardiograms were recommended by the ID consultant team. Lastly, because of the small number of patients in our study, we had limited power to detect significant differences between those with and without ID consultation.

## CONCLUSIONS

Although we did not find a significant difference, the lower mortality in patients who had ID consultation suggests that it should be required in all patients with *S. aureus* bacteremia, including those at low risk for endocarditis and who have no secondary infection. Additionally, adherence to guidelines regarding antimicrobial choice and route of administration, as well as follow-up blood cultures, was variable in patients without ID consultation, lending further support to requiring ID consultation in this patient population.

## APPENDIX

Table A1. Cause of Death With ID Consult (n = 5)

**Table:**

In-Hospital COD	Related to SAB	30-d COD	Related to SAB	90-d COD	Related to SAB
Septic shock	Yes	Septic shock due to UTI	No	Liver failure	No
Septic shock	Yes				
Mixed shock	Yes				

Abbreviations: COD, cause of death; SAB, *Staphylococcus aureus* bacteremia; UTI, urinary tract infection.

**Table A2. Cause of Death Without ID** **Consult (n = 9**)

**Table:**

In-hospital COD	Related to SAB	30-d COD	Related to SAB	90-d COD	Related to SAB
Hemorrhagic stroke^a^	No	Septic shock (hospice)	Yes	Pneumonia	Yes
Hemorrhagic stroke^a^	No	Septic shock (hospice)	Yes		
Septic shock	Yes				
Septic shock	Yes				
Septic shock	Yes				
Alcoholic hepatitis	No				

Abbreviations: COD, cause of death; SAB, *Staphylococcus aureus* bacteremia.

aAll hemorrhagic strokes occurred 72 or more hours prior to the time first positive blood cultures were obtained.
